# Comparative Effects of Structured Yoga and Conventional Therapeutic Exercise on Pain and Disability in Chronic Mechanical Low Back Pain: A Short-Term Randomized Study

**DOI:** 10.7759/cureus.101636

**Published:** 2026-01-15

**Authors:** Ajay Bharti, Vivek Kumar, Sanjay Kumar, Rajnand Kumar, Nitish Kumar, Sudhir Shyam Kushwaha

**Affiliations:** 1 Orthopedic Surgery, All India Institute of Medical Sciences, Gorakhpur, IND; 2 Orthopedic Surgery, Ganesh Shankar Vidyarthi Memorial Medical College, Kanpur, IND

**Keywords:** chronic low back pain (clbp), conventional therapeutic exercise (cte), functional disability, hatha yoga, pain intensity, structured yoga

## Abstract

Background

Chronic mechanical low back pain (CMLBP) is a common cause of disability, and exercise-based interventions are first-line management. Yoga has emerged as a mind-body alternative, but evidence comparing yoga with conventional therapeutic exercise (CTE) remains inconsistent.

Objective

This study aims to compare the short-term effects of a structured yoga program versus CTE on pain intensity, analgesic use, and functional disability in adults with CMLBP.

Methods

A single-center, parallel-group randomized, comparative study was conducted in a tertiary care center in North India. Sixty participants (ages 25-40 years) with CMLBP were randomized (1:1) to either yoga (25-minute supervised sessions twice weekly + home practice) or CTE (50-minute supervised daily exercise) for six weeks. Primary outcomes were pain intensity (visual analog scale (VAS)) and weekly analgesic consumption. The secondary outcome was functional disability (Oswestry Disability Index (ODI)). Analyses followed the intention-to-treat principle. Between-group comparisons used independent t-tests and analysis of covariance (ANCOVA) (adjusting for baseline). Clinical significance was evaluated using established minimal clinically important difference (MCID) thresholds (VAS ≥ 1.5-2.0; ODI ≥ 10).

Results

All 60 participants completed the study (adherence: ≥90%). Both groups demonstrated significant within-group improvements in VAS, ODI, and analgesic use (all p < 0.001; large effect sizes d = 2.02-3.82). Between-group analysis favored yoga for all outcomes: VAS reduction showing yoga 4.26 vs. CTE 3.60 (t = 2.78, p = 0.007; d = 0.71, adjusted mean difference = 0.6 points; 95% CI 0.20-1.06); ODI reduction showing yoga 21.7 vs. CTE 17.2 (t = 3.21, p = 0.002; d = 0.82; adjusted mean difference = 4.5, 95% CI 1.73-7.67); and analgesic reduction showing yoga 7.9 vs. CTE 6.8 (t = 2.21, p = 0.031; d = 0.56, adjusted mean difference = 1.0 tablet/week, 95% CI 0.3-2.3), not significant after Bonferroni correction (α = 0.0167). Between-group differences for VAS (≈0.6) and ODI (≈4.5) did not exceed MCID, indicating clinically modest advantages despite statistical significance. No serious adverse events occurred.

Conclusion

A short-duration, structured yoga program resulted in greater short-term improvement in pain, disability, and analgesic use than a higher-dose CTE regimen, although the clinical superiority was modest. Yoga may represent a time-efficient, low-burden alternative, rather than a definitively superior intervention. Larger, multicenter trials with long-term follow-up and dose-matched protocols are warranted.

## Introduction

Chronic low back pain (CLBP) is prevalent globally, with a point prevalence of 7.3%, and is a major contributor to disability and diminished quality of life [1,2]. Most cases are categorised as nonspecific low back pain (mechanical low back pain) as they lack a defined pathoanatomical cause, which makes diagnosis and management challenging [3]. Accessory ossicles are often asymptomatic and found incidentally; however, some, such as the limbus vertebrae, can also contribute to nonspecific low back pain. Therefore, understanding the anatomical variations and prevalence of these ossicles is crucial for accurate diagnosis and targeted treatment strategies in patients with chronic or nonspecific low back pain, helping to reduce the global disease burden associated with CLBP [4]. Management focuses on symptom relief, functional restoration, and disability mitigation through education, reassurance, analgesics, and nonpharmacological therapies. While many patients improve with minimal intervention, overuse of imaging, opioids, injections, and surgery has escalated dramatically in recent decades, often without demonstrable improvements in outcomes, highlighting gaps between guideline recommendations and real-world practice. Current strategies emphasize risk stratification, stepped care, and individualized treatment to maximize benefits and minimize harm, yet the increasing complexity and commercialization of chronic back pain interventions highlight the need for evidence-based, scalable, and pragmatic approaches to care [3,5]. Evidence-based guidelines recommend nonpharmacological therapies, particularly exercise, mind-body interventions such as yoga and mindfulness, and multidisciplinary rehabilitation, as first-line treatments, with medications reserved for nonresponders.

Meta-analytic data indicate that strength/resistance and coordination/stabilization exercises effectively reduce pain and disability, whereas other exercise modalities show limited benefit. These findings highlight the importance of using structured, evidence-based exercise and mind-body strategies in managing CLBP, along with the necessity for interventions that are both effective and feasible in everyday practice care [6,7].

Yoga is increasingly recognized as a safe, nonpharmacological approach for CLBP, integrating postures, breathing (pranayama), and mindfulness to enhance trunk strength, flexibility, postural control, and stress modulation. Theoretical frameworks suggest that yoga’s asanas and breathing techniques engage isometric muscle activation and mind-body integration to improve physical and emotional harmony [8]. Although some studies report benefits comparable to conventional exercise [9], systematic reviews indicate that yoga can reduce pain and disability, with stronger effects in the short term. However, optimal dosing, intensity, and its comparative effectiveness relative to conventional therapeutic exercise remain unclear. Most previous trials have used long-duration sessions (60-90 minutes), limiting understanding of whether shorter, more feasible protocols can achieve similar outcomes, an important issue for real-world implementation [10,11]. Additionally, conventional exercise programs vary widely, and few studies have compared yoga to a standardized physiotherapy regimen. Prior work, such as the trial by Neyaz et al. [12], showed that hatha yoga produced improvements similar to conventional exercises, but that study used longer-duration protocols and did not assess a condensed, time-efficient format. Thus, it remains uncertain whether short, structured yoga sessions can provide meaningful benefits relative to a more intensive exercise program, constituting the primary research gap addressed by this study. Accordingly, the present trial evaluates the short-term effectiveness of a structured hatha yoga program compared with a standardized conventional therapeutic exercise regimen in adults with CLBP. Therefore, the primary objective was to compare the short-term effects of a twice-weekly structured yoga program with those of a standardized conventional therapeutic exercise regimen on pain intensity and weekly analgesic use in adults with chronic mechanical low back pain (CMLBP). Secondary objectives were to compare the impact on functional disability and to evaluate the safety, adherence, and feasibility of a time-efficient yoga protocol. We hypothesized that the yoga group would demonstrate greater reductions in pain intensity and functional disability over six weeks.

## Materials and methods

This prospective, randomized, longitudinal study was conducted jointly in the departments of orthopedics and yoga at a tertiary care center in North India over a one-year period (August 1, 2023, to August 1, 2024). A total of 60 participants with CMLBP were recruited and randomly assigned into two equal groups: a yoga intervention group and a CTE group. This trial was designed as an exploratory randomized study. The sample size was chosen based on feasibility and available resources rather than an a priori power calculation. The randomization process involved generating random sequences using computer-based random numbers. Opaque, sealed, sequentially numbered envelopes were utilized for allocation concealment. An independent assistant performed the group assignment. Due to the nature of the interventions, participant blinding was not feasible.

Eligible participants were men and women aged 25-40 years with low back pain persisting for at least 12 weeks and reporting a minimum pain intensity of 4 on a 10-point visual analog scale (VAS). Individuals with prior spinal surgery, participation in other structured exercise programs, neurological deficits or radiculopathy, specific spinal pathologies (such as infection, fracture, malignancy, or inflammatory conditions), pregnancy, or medical contraindications to exercise were excluded. Written informed consent was obtained from all participants before enrolment.

The yoga group underwent structured hatha yoga therapy for six weeks. The structured hatha yoga program consisted of twice-weekly, 25-minute, supervised group sessions for six weeks. The protocol was designed and delivered by a certified yoga therapist with extensive experience in managing musculoskeletal conditions. Each session followed a standardized sequence: an introductory period of education, breathing exercises (pranayama), subtle warm-up movements (sukshma vyayama), a series of strengthening and stretching postures (asanas), and a final guided relaxation (Table 1). The program was designed to enhance proprioception, core stability, and mindfulness. The yoga instructor ensured correct alignment for all participants and provided individualized guidance on the intensity of each pose within the structured timeframe to prioritize safety and proper form. The aim was to perform each movement with mindful awareness rather than to achieve maximum range of motion.

The CTE protocol was based on established principles of core stabilization for mechanical low back pain. The daily, 50-minute sessions were administered by a licensed clinical practitioner under the supervision of an orthopaedician. The program was designed to enhance lumbar stability and neuromuscular control while educating participants on movements to avoid symptom exacerbation (Table 2). The difficulty of exercises was progressed based on individual participant tolerance and improvement.

Intervention adherence was monitored separately for supervised and self-practice sessions. For the CTE group, adherence was calculated as the proportion of 42 scheduled supervised sessions attended. For the yoga group, adherence was combined with attendance at 12 supervised sessions and self-reported completion of 30 home-practice sessions recorded in participant diaries. Adherence percentage was computed as [(Sessions completed ÷ Sessions prescribed) \times 100].

A threshold of ≥90% session completion was defined as “excellent adherence,” consistent with adherence standards for behavioral and therapeutic exercise interventions recommended in the TIDieR framework and supported by behavioral intervention literature [13,14].

Outcomes, assessed at baseline and six weeks, included pain intensity (VAS), disability (Oswestry Disability Index (ODI)) [15], and weekly analgesic consumption. The VAS and weekly analgesic consumption were primary outcome measures, while the ODI was a secondary outcome measure. Outcome assessment relied on self-reported measures (VAS and ODI), and assessors were not blinded to group allocation. Ethics approval was obtained from the Institutional Human Ethics Committee (IHEC/AIIMS-GKP/BMR/150/2023).

The normality of continuous data was assessed using the Shapiro-Wilk test. Continuous variables are presented as mean ± standard deviation (SD) and categorical variables as counts and percentages. For each comparison, test statistics, p-values, 95% confidence intervals (CIs), and effect sizes were reported to evaluate both statistical and clinical significance. Cohen’s d was used for continuous variables, and the Phi coefficient (φ) for categorical variables. Baseline characteristics of participants in the CTE and yoga groups were compared using independent sample t-tests for continuous variables (e.g., age, duration of symptoms, weekly analgesic dose) and chi-square tests for categorical variables (e.g., sex, marital status, occupation). Within-group changes in VAS score, ODI, and weekly analgesic dose were assessed using paired t-tests to evaluate intervention effects over time. Between-group differences in treatment efficacy were analyzed using independent samples t-tests on change scores (post-treatment minus baseline values) for VAS, ODI, and weekly analgesic dose. Cohen’s d was calculated to estimate effect size, interpreted as small (d = 0.2), medium (d = 0.5), or large (d ≥ 0.8). All tests were two-tailed, with statistical significance set at p < 0.05.

Clinically meaningful improvement was assessed based on established minimal clinically important difference (MCID) values for CLBP (approximately 1.5-2 points for VAS [16,17] and 10 points for ODI [18,19]. These thresholds were used to contextualize effect sizes and CIs in the interpretation of results. Between-group comparisons for the three primary outcomes (VAS, weekly analgesic consumption, ODI) were adjusted for multiple testing using Bonferroni correction, yielding an adjusted significance threshold of α = 0.0167 (0.05/3). Within-group comparisons were considered exploratory and therefore not corrected. As VAS and analgesic consumption were designated as primary outcomes, between-group comparisons were further examined using analysis of covariance (ANCOVA), with baseline scores as covariates and age and duration of symptoms as adjustment variables, given their borderline imbalance at baseline (p = 0.073 and p = 0.087). ODI, a secondary outcome, was analyzed with and without adjustment to assess consistency. Analyses were conducted in MS Excel (Microsoft Corporation, Redmond, Washington, United States) and cross-checked using IBM SPSS Statistics for Windows, Version 28 (Released 2021; IBM Corp., Armonk, New York, United States) to enhance computational reliability.

An adverse event (AE) was defined as any undesirable or unintended sign, symptom, or medical occurrence that developed or worsened during participation in the intervention, regardless of its causal relationship to the study. Participants were specifically asked about worsening of pain, musculoskeletal discomfort, dizziness, fatigue, or other new symptoms at each supervised session and follow-up. Serious adverse events (SAEs), such as hospitalization, life-threatening events, or major injuries, must be immediately reported to the ethics committee. All AEs and withdrawals were documented in standardized case report forms. The final interpretation of all analytical outputs was conducted by the authors.

**Table 1:** Protocol for structured yoga intervention VAS: visual analog scale Note: The pose listed as "Ustharasana" in the original protocol refers to Setu Bandhasana (bridge pose). This pose was selected for its focus on gentle spinal extension and strengthening of the gluteal and hamstring muscles

Component	Specific exercises	Duration & dosage	Description/ purpose
Session frequency		Twice weekly for 6 weeks	Supervised group sessions
Total session time		25 minutes	
Introduction & education	Benefits of yoga, correct posture	6 minutes	Education on the role of mind-body interventions for pain management
Breathing (Pranayama)	Nadi Shodhana (alternate nostril breathing)	2 minutes	Practiced to promote relaxation and breath awareness
Warm-up (Sukshma Vyayama)	Kati Chakrasana (waist rotations), Tarasana (palm tree pose)	4 minutes	Gentle movements to prepare the spine and muscles
Main poses (Asanas)	Salabhasana (locust pose), Bhujangasana (cobra pose) Dhanurasana (bow pose), Setu Bandhasana (bridge pose)*	8 minutes	Poses focused on gentle spinal extension and strengthening of the posterior chain
Relaxation	Savasana (corpse pose)	5 minutes	Guided relaxation to integrate the benefits of the practice
Modifications/contraindications			Postures modified for pain >5/10 VAS, radiculopathy, dizziness; immediate cessation if neurological symptoms arise
Safety & alignment principles			Neutral spine positioning; avoidance of end-range lumbar flexion/extension; pain-free range only; individual modifications provided; props used as needed

**Table 2:** Protocol for CTE CTE: conventional therapeutic exercise Note: Exercises were progressed weekly by increasing repetitions, hold duration, or exercise complexity based on participant tolerance

Component	Specific exercises	Duration & dosage	Description/purpose
Session frequency		Daily for 6 weeks	Daily supervised sessions by a physiotherapist
Total daily time		~50 minutes	
Education & advice	Avoid forward bending, squatting; use lumbar support while sitting	Integrated throughout	Education on spinal hygiene and ergonomics to prevent aggravation
Core stabilization	Transversus abdominis activation: quadruped arm/leg raises (bird-dog), dead bug exercise, glute bridges, side-lying clamshells	25-30 minutes, 3 sets of 10-15 repetitions	Focus on controlled movement, core bracing, and maintaining a neutral spine
Spinal extension & mobility	Prone lying (progressing to propping on elbows) McKenzie press-ups	15-20 minutes, 10 repetitions, held for 5-10 seconds at the top	Performed pain-contingent (stopped if pain radiated)
Cool-down/stretching	Hamstring stretch piriformis stretch	5 minutes. Each stretch held for 30 seconds, repeated 2-3 times	
Contraindications/modifications			Avoid end-range movements provoking radicular pain, dizziness, or increased stiffness
Safety and alignment principles			Risk during progressive loading or incorrect posture

## Results

**Figure 1:**
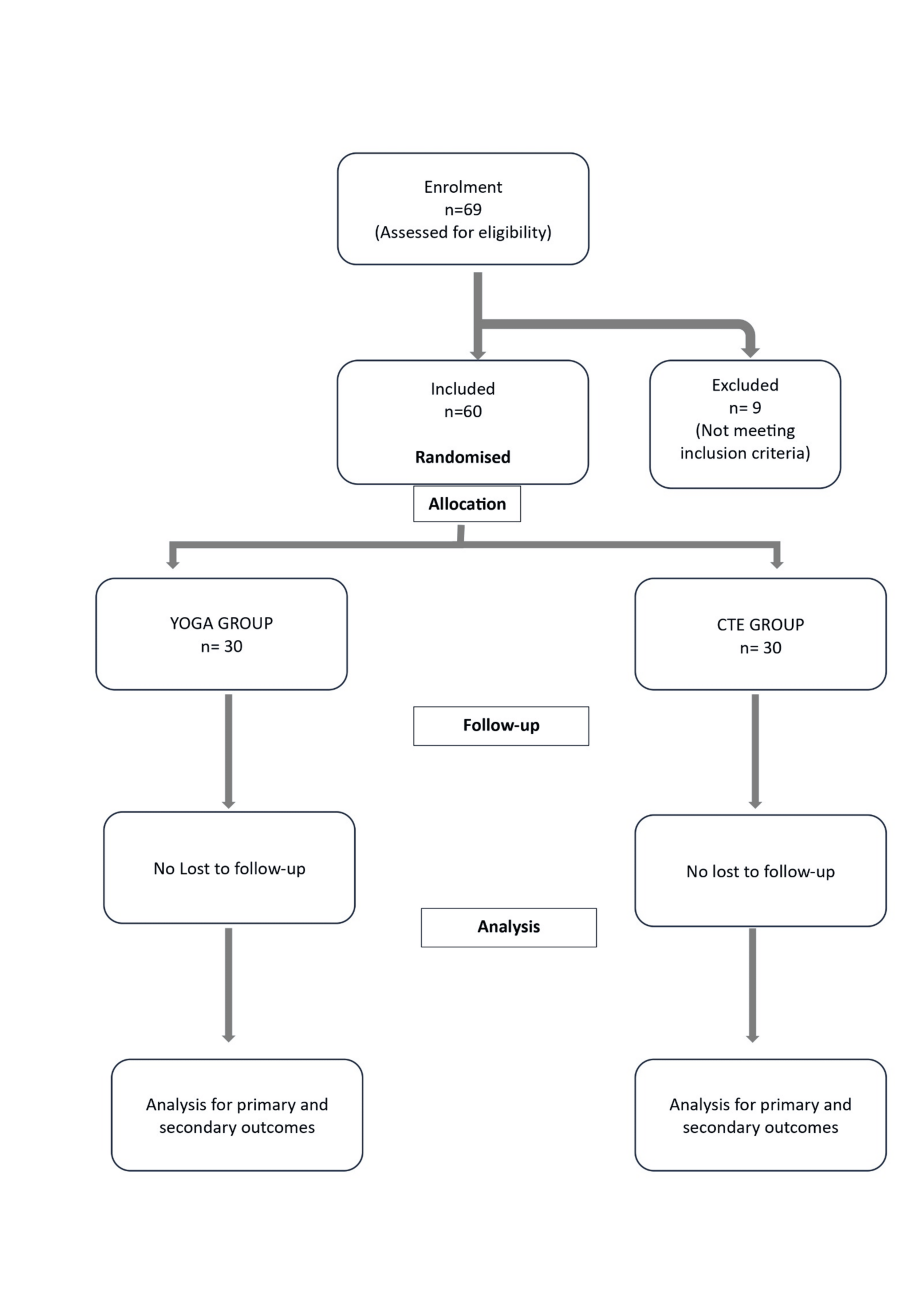
Consort chart for participant flow

The study was conducted as a pilot study and was registered in our institute's ethical committee. Participant flow is illustrated in Figure 1.

Intervention adherence was excellent across both treatment groups. Participants in the CTE group attended a mean of 38.1 ± 3.5 out of 42 sessions (90.7%), while those in the yoga group completed 11.1 ± 1.0 supervised sessions (92.5%) and 27.4 ± 2.8 self-practice sessions (91.3%), giving an overall adherence of 91.7%. No replacement, withdrawal, or imputation procedures were required. Therefore, intervention adherence exceeded 90%, and no AEs were reported. This high level of compliance indicates that both intervention protocols were feasible and well-accepted. All analyses adhered to the intention-to-treat (ITT) principle. Since there were no dropouts or missing outcome data, no imputation was necessary. Although ITT was planned, no participants were lost to follow-up; therefore, ITT and per-protocol analyses were identical. ITT and per-protocol analyses yielded the same sample (n = 60), including all 60 randomized participants (30 per group) in their original groups. Between-group comparisons of post-intervention VAS and ODI scores were performed using ANCOVA with baseline scores as covariates. Within-group changes from baseline were assessed via paired t-tests. Effect sizes (Cohen’s d) were calculated for each outcome to determine the extent of improvement.

All 60 participants completed the interventions. The baseline demographic and clinical characteristics of participants in the CTE and yoga groups were comparable, with no statistically significant differences observed between groups across any variable (all p > 0.05). Confidence intervals for continuous variables included zero, and effect sizes were small to moderate. These details are provided in Table 3.

**Table 3:** Baseline demographic and clinical characteristics of participants CI: confidence interval; effect sizes: Cohen’s d for t-tests; Phi coefficient (φ) for chi-square tests Values are presented as mean ± standard deviation for continuous variables and counts (percentages) for categorical variables

Variable	CTE group (n = 30)	Yoga group (n = 30)	Test used	Test statistic	p-value	Lower 95% CI	Upper 95% CI	Effect size
Age (years)	30.2 ± 4.3	32.1 ± 4.6	Independent t-test	t = -1.82	0.073	−4.02	0.18	d = 0.43
Sex	16 / 14	17 / 13	Chi-square test	χ² = 0.07	0.791	---	---	φ = 0.03
Marital status	20 / 10	21 / 9	Chi-square test	χ² = 0.10	0.752	---	---	φ = 0.04
Occupation (employed)	22 (73.3%)	24 (80%)	Chi-square test	χ² = 0.29	0.589	---	---	φ = 0.07
Duration of symptoms (months)	11.6 ± 5.3	13.9 ± 5.8	Independent t-test	t = -1.74	0.087	−4.96	0.34	d = 0.41

Although baseline characteristics were largely comparable between groups, age and duration of symptoms approached borderline significance (p = 0.073 and p = 0.087), with small-to-moderate effect sizes (d = 0.43 and d = 0.41). In a sample of only 30 participants per group, such nonsignificant differences may still have practical clinical relevance and introduce residual confounding. To account for this, adjusted analyses were performed using ANCOVA with baseline values as covariates.

**Figure 2:**
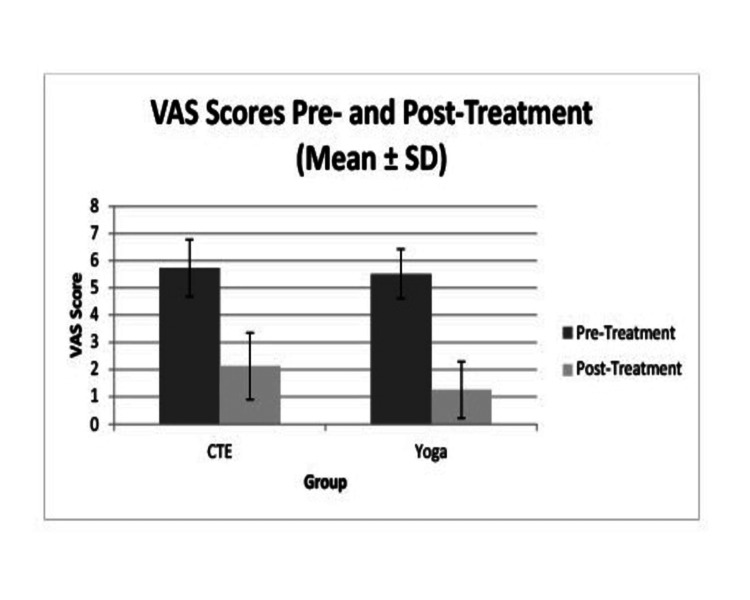
Comparison of VAS scores between the two groups SD: standard deviation; VAS: visual analog scale

At baseline, there were no statistically significant differences in VAS scores between the CTE and yoga groups (p > 0.05), indicating comparable levels of pain prior to intervention. Both groups demonstrated statistically significant reductions in VAS scores from baseline to six weeks with the CTE group showing a mean VAS decreased from 5.73 ± 1.05 to 2.13 ± 1.23, yielding a mean difference of 3.60 (t = 15.82, p < 0.001, Cohen’s d = 2.89) and the yoga group showing that the mean VAS decreased from 5.52 ± 0.91 to 1.26 ± 1.04, with a mean difference of 4.26 (t = 20.94, p < 0.001, Cohen’s d = 3.82). An independent samples t-test on the change scores showed a statistically significant difference (t = 2.78, p = 0.007) with a moderate effect size (Cohen’s d = 0.71). The 95% confidence interval for the between-group difference ranged from 0.20 to 1.06, indicating clinical relevance. These results suggest that both interventions effectively reduced pain intensity, but the yoga intervention demonstrated superior efficacy over the six-week period (Table 4, Figure 2).

**Table 4:** Comparison of VAS scores pre- and posttreatment between CTE and yoga groups VAS: visual analog scale; CTE: conventional therapeutic exercise; CI: confidence interval; effect size: Cohen’s d VAS scores range from 0 (no pain) to 10 (worst pain imaginable)

Group	VAS pretreatment (mean ± SD)	VAS posttreatment (mean ± SD)	Mean difference	Lower 95% CI	Upper 95% CI	Test used	Test statistic	p-value	Effect size
CTE	5.73 ± 1.05	2.13 ± 1.23	3.60	3.23	3.97	Paired t-test	t = 15.82	<0.001	d = 2.89
Yoga	5.52 ± 0.91	1.26 ± 1.04	4.26	3.92	4.60	Paired t-test	t = 20.94	<0.001	d = 3.82
Between-group	-	-	-	0.20	1.06	Independent t-test	t = 2.78	0.007	d = 0.71

After adjustment for baseline VAS, age, and duration of symptoms using ANCOVA, the between-group difference favored the yoga group (adjusted mean difference = 0.6 points; 95% CI 0.20-1.06; p = 0.007). However, this improvement did not exceed the MCID threshold (≥1.5-2.0 points), indicating a statistically significant but clinically modest effect.

**Figure 3:**
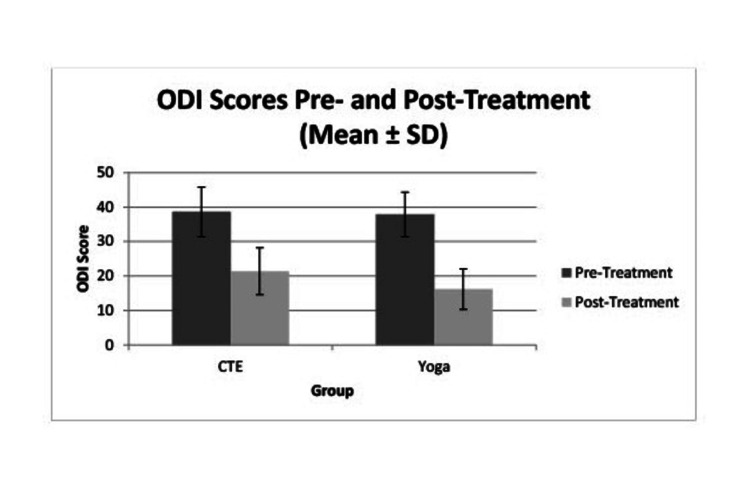
Comparison of ODI scores after intervention in both the groups ODI: Oswestry Disability Index; SD: standard deviation

At baseline, ODI scores were comparable between the CTE and yoga groups (p > 0.05), indicating similar levels of functional disability prior to intervention. Both groups showed statistically significant improvements in ODI scores from baseline to six weeks. ODI scores improved significantly in both groups. The CTE group showed a reduction from 38.6 ± 7.2 to 21.4 ± 6.8 (mean change = 17.2; p < 0.001; Cohen’s d = 2.37), while the yoga group improved from 37.9 ± 6.5 to 16.2 ± 5.9 (mean change = 21.7; p < 0.001; Cohen’s d = 3.00). After adjusting for baseline ODI scores using ANCOVA, between-group comparison of change scores demonstrated a significantly greater reduction in the yoga group (adjusted mean difference = 4.5 points; 95% CI 1.73-7.67; p = 0.002), with a moderate-to-large effect size (Cohen’s d = 0.82). These findings suggest that both interventions effectively reduced disability, but yoga produced a more pronounced improvement in functional outcomes over the six-week period (Table 5, Figure 3).

**Table 5:** Comparison of ODI scores pre- and posttreatment between CTE and yoga groups ODI: Oswestry Disability Index; CI: confidence interval; effect size: Cohen’s d ODI scores range from 0 (no disability) to 100 (maximum disability)

Group	ODI pretreatment (mean ± SD)	ODI posttreatment (mean ± SD)	Mean difference	Lower 95% CI	Upper 95% CI	Test used	Test statistic	p-value	Effect size
CTE	38.6 ± 7.2	21.4 ± 6.8	17.2	14.8	19.6	Paired t-test	t = 13.47	<0.001	d = 2.37
Yoga	37.9 ± 6.5	16.2 ± 5.9	21.7	19.3	24.1	Paired t-test	t = 17.03	<0.001	d = 3.00
Between-group	—	—	—	1.73	7.67	Independent t-test	t = 3.21	0.002	d = 0.82

Both groups achieved improvements exceeding the established MCID for ODI (≥10 points), indicating clinically meaningful benefit in each group. Although statistically significant, the between-group difference did not exceed the established MCID of 10 points, indicating that both interventions produced clinically meaningful improvement, while yoga conferred a modest additional benefit.

**Figure 4:**
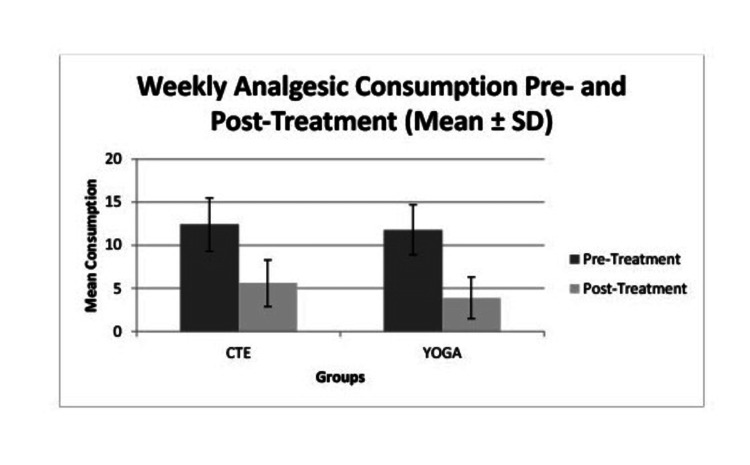
Comparison of weekly analgesic consumption between two groups SD: standard deviation

Both interventions led to significant reductions in weekly analgesic consumption. In the CTE group, the mean reduction was 6.8 tablets per week, with a 95% confidence interval of 5.9 to 7.7, demonstrating a large effect size (Cohen’s d = 2.02, p < 0.001). Similarly, the yoga group showed a mean reduction of 7.9 tablets per week, with a 95% confidence interval of 7.0 to 8.8 and a large effect size (Cohen’s d = 2.55, p < 0.001). The between-group comparison using an independent samples t-test showed a mean difference of 1.1 tablets per week, favoring the yoga group, which was statistically significant (t = 2.21, p = 0.031) with a moderate effect size (Cohen’s d = 0.56) (Table 6, Figure 4).

**Table 6:** Comparison of weekly analgesic consumption pre- and posttreatment between CTE and yoga groups CI: confidence interval; effect size: Cohen’s d Weekly analgesic consumption expressed in tablets/week

Groups	Weekly analgesic consumption pretreatment (mean ± SD)	Weekly analgesic consumption posttreatment (mean ± SD)	Mean reduction	Lower 95% CI	Upper 95% CI	Test used	Test statistic	p-value	Effect size
CTE	12.4 ± 3.1	5.6 ± 2.7	6.8	5.9	7.7	Paired t-test	t = 10.42	<0.001	d = 2.02
YOGA	11.8 ± 2.9	3.9 ± 2.4	7.9	7.0	8.8	Paired t-test	t = 13.17	<0.001	d = 2.55
Between-group	-	-	-	0.3	2.3	Independent t-test	t = 2.21	0.031	d = 0.56

**Figure 5:**
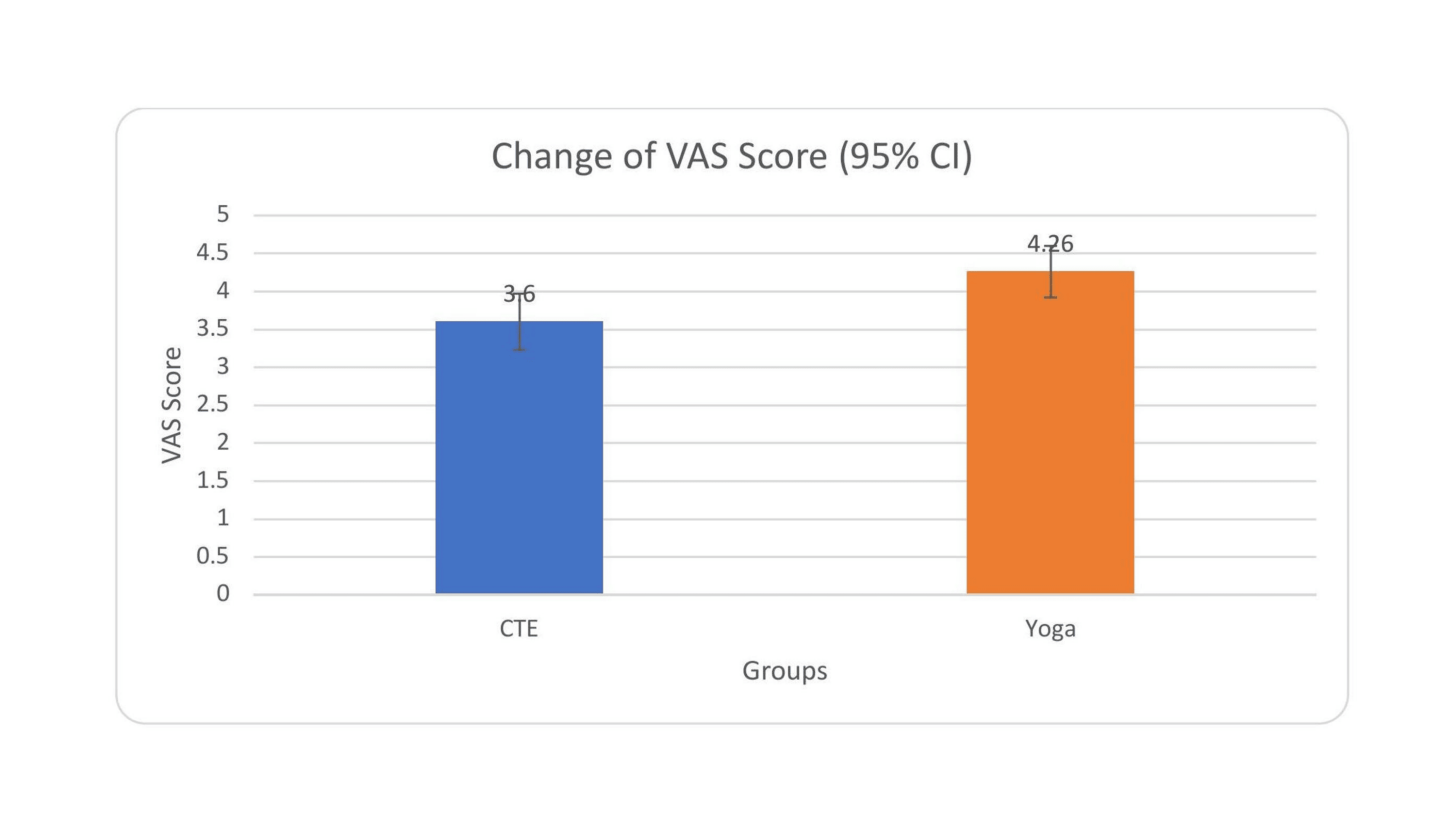
Improvement in VAS scores following intervention with 95% CI VAS: visual analog scale; CI: confidence interval Error bars represent 95% CI

**Figure 6:**
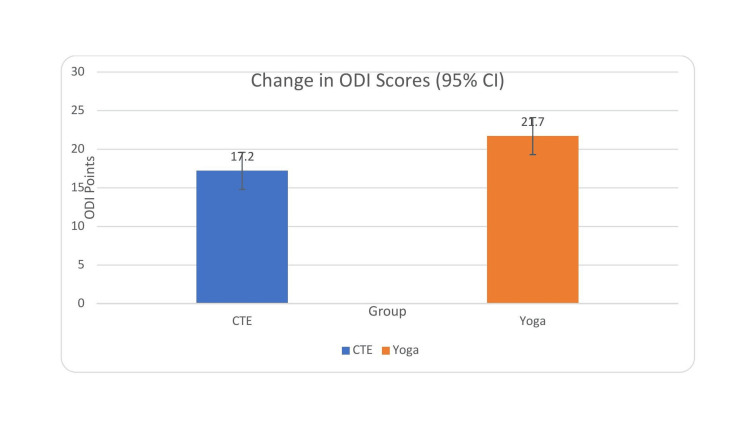
Change in Oswestry Disability Index (ODI) scores from baseline to post-intervention with 95% CI CI: confidence interval Error bars represent 95% CIs

**Figure 7:**
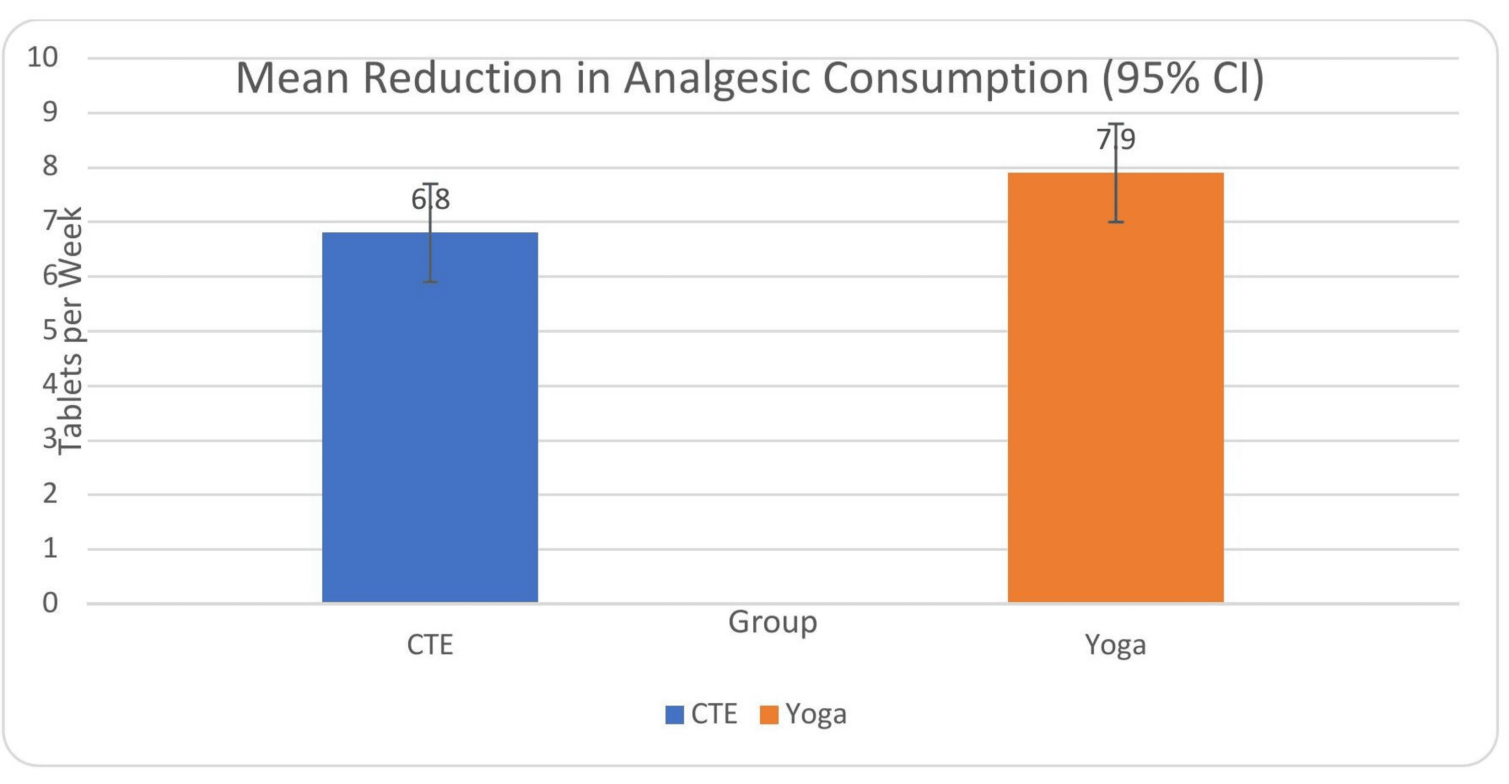
Mean reduction in weekly analgesic consumption following intervention with 95% CI CI: confidence interval Error bars represent 95% CIs

However, after adjustment for baseline analgesic use, age, and duration of symptoms using ANCOVA, the between-group difference was no longer statistically significant (adjusted mean difference = 1.0 tablet/week, 95% CI 0.3-2.3; p = 0.093 (after Bonferroni correction)), indicating that the reduction in analgesic use should be interpreted cautiously. Comparison of change in pain scores (VAS), disability scores (ODI), and weekly analgesic consumption after six weeks of yoga or CTE intervention, with 95% confidence intervals, has been depicted in Figures 5-7.

To avoid residual confounding resulting from small sample size, adjusted analyses were performed using ANCOVA with baseline values as covariates; these adjustments did not materially change the direction or statistical significance of the primary outcomes, suggesting that the findings were robust to baseline variation. However, the modest absolute between-group differences (VAS ≈0.6; ODI ≈4.5) remained below MCID thresholds despite adjustment, indicating that any advantage of yoga should be interpreted as clinically modest rather than decisively superior. Future trials should consider stratified randomization or larger, adequately powered samples to minimize imbalance and improve the certainty of estimates.

After Bonferroni correction (adjusted α = 0.0167), between-group differences remained statistically significant for VAS (p = 0.007) and ODI (p = 0.002), while the difference in analgesic consumption did not retain significance (p = 0.031 ≥ 0.0167). All within-group improvements remained significant (all p < 0.001). Both interventions produced significant short-term improvements; however, the magnitude of change was greater in the yoga group across all primary outcomes. VAS scores improved with very large within-group effect sizes (CTE: d = 2.89; yoga: d = 3.82), ODI decreased with huge effect sizes (CTE: d = 2.37; yoga: d = 3.00), and weekly analgesic consumption declined substantially (CTE: d = 2.02; yoga: d = 2.55). Between-group differences favored yoga with moderate-to-large effects for VAS (d = 0.71) and ODI (d = 0.82), although the difference in analgesic use showed only a moderate effect (d = 0.56) and did not remain statistically significant after Bonferroni correction.

## Discussion

In this study, both yoga and CTE produced significant short-term improvements in pain (VAS), disability (ODI), and analgesic use in adults with CMLBP. Yoga demonstrated greater mean reductions across all outcomes; however, between-group differences were clinically modest, and superiority was not consistently maintained after statistical adjustment. These findings suggest that yoga may represent a lower-burden alternative rather than a definitively superior intervention. The high adherence observed (>90%) underscores the practicality and acceptability of both interventions in a real-world rehabilitation setting. Such levels of compliance are rarely achieved in long-duration exercise trials, highlighting the strength of participant engagement and fidelity of intervention delivery.

Statistically significant findings alone risk overinterpretation; therefore, clinical meaning was evaluated using MCID thresholds (VAS ≥ 1.5-2.0; ODI ≥ 10). Both groups exceeded MCID values, confirming patient-perceived benefit. Between-group differences did not exceed MCID (VAS ≈0.6; ODI ≈4.5 points), indicating that while yoga outperformed CTE statistically, the magnitude of difference was not large enough to be considered clinically decisive. For analgesic use, the initial between-group significance (p = 0.031) did not withstand Bonferroni correction (α = 0.0167) and should therefore be interpreted with caution. Therefore, the magnitude of improvement in pain and disability exceeded established MCID thresholds, suggesting that the observed effects were not only statistically significant but also clinically meaningful. The consistently large within-group effect sizes observed for VAS, ODI, and analgesic use suggest meaningful short-term clinical improvement, particularly in the yoga group. However, the magnitude of these effects (e.g., VAS d = 3.82; ODI d = 3.00) is unusually high for clinical trials and may partially reflect expectancy effects, self-report bias, high adherence, and the small sample size. This further highlights the need for dose-matched, adequately powered trials to determine whether the observed differences are sustainable and reproducible. Between-group effect sizes were more modest (VAS d = 0.71; ODI d = 0.82; analgesics d = 0.56), indicating that while yoga may confer incremental benefit over conventional exercise, the superiority should be interpreted cautiously, especially as the analgesic difference did not retain significance after Bonferroni correction.

The greater short-term improvement observed with yoga may relate to its broader mind-body integration compared with CTE. The yoga program combined physical postures, breathing control, relaxation, and mindful awareness-elements known to enhance parasympathetic activity, reduce sympathetic arousal, and modulate central sensitization through stress-response regulation [20]. These physiological effects, together with improved body awareness and cognitive reframing, can lessen fear-avoidant behavior, catastrophizing, and perceived disability, thereby enhancing self-efficacy and pain coping. In contrast, the higher-dose CTE program emphasized biomechanical reconditioning through strengthening and stretching but did not specifically address autonomic or psychosocial domains. Consequently, yoga’s effects may reflect multidimensional modulation, physiological, psychological, and behavioral, rather than purely physical mechanisms [10,21]. However, given the unequal intervention dosage and lack of long-term follow-up, these interpretations remain exploratory and warrant confirmation in dose-matched, mechanistic studies.

Our findings partially align with existing literature on yoga for chronic low back pain. A recent Cochrane review of 21 randomized trials (n > 2200) concluded that yoga produces small, clinically modest improvements in pain and function compared with no exercise and offers little to no superiority over conventional exercise-based rehabilitation (9). In contrast, the present study observed larger short-term improvements with yoga relative to supervised therapeutic exercise. This discrepancy may reflect several factors: our structured protocol, higher adherence (>90%), and the comparatively younger cohort, as well as the unequal intervention dosage between groups. Therefore, our results should not be interpreted as evidence of intrinsic superiority, but rather as an indication that a lower-burden, structured yoga program may be a feasible and efficient alternative for some patients.

In contrast to trials comparing yoga with generic exercise, studies directly evaluating yoga against stabilization or core-focused programs report similar functional outcomes. Ulger et al. found that both interventions improved pain and disability (VAS, ODI) comparably, with stabilization showing only slightly greater gains in core activation [22]. Likewise, Desai et al. reported differences in neuromuscular activation patterns but no clear clinical superiority of either modality [23]. Collectively, these findings align with our results, indicating that yoga may be as effective as stabilization-based therapy in the short term, rather than consistently outperforming it when intervention intensity and exposure are comparable.

This study has certain limitations. First, the imbalance in intervention duration (yoga ≈50 min/week vs. CTE ≈350 min/week) introduces a confounding dosage effect, preventing isolation of treatment-specific efficacy. This presents a possible confounding variable, in which differences in outcomes might be driven by treatment dose and participant burden rather than by the intrinsic therapeutic effectiveness of yoga. Second, the absence of blinding for participants and assessors increases the risk of performance and detection bias, particularly given reliance on self-reported measures. Third, since chronic low back pain is a long-term and often recurring condition, the short six-week follow-up limits interpretation to immediate outcomes; the durability of these improvements and the risk of relapse or ongoing benefits cannot be confirmed. Fourth, the single-center design and restricted age range (25-40 years) limit generalizability. A larger, more diverse cohort would improve the precision of effect estimates and enhance external validity. Although analyses were cross-checked in SPSS, initial use of Excel is recognized as a methodological constraint.

All analyses were conducted using the intention-to-treat approach, and since all randomized participants completed the study, the ITT and per-protocol populations were identical, eliminating attrition bias. This high completion rate strengthens internal validity and supports the feasibility of both interventions. Future studies with larger samples and longer follow-up should continue using ITT to enhance comparability and reproducibility. No serious adverse events occurred, and adherence exceeded 90% in both groups, supporting feasibility, close supervision, structured progression, individualized modification, and short-term safety. However, yoga was associated with higher adverse event rates, as shown in previous trials by Wieland et al. and Sherman et al. Therefore, due to yoga-related symptom exacerbation in real-world settings, safe implementation requires supervision, individualized modification, and attention to tolerance and contraindications [9,24].

Yoga may serve as a feasible and lower-burden option for guideline-based management of CMLBP, particularly where time or access to supervised therapy is limited. This study measured pain, disability, and analgesic use only. Broader yoga-related outcomes, such as quality of life, psychological well-being, coping, and mindfulness, as well as long-term follow-up, were not assessed, limiting insight into broader biopsychosocial effects. Larger, dose-matched, blinded, and adequately powered multicentered trials with longer follow-up and multidimensional outcomes (e.g., quality of life, psychological distress, coping, occupational participation) are required to determine whether yoga provides sustained, clinically meaningful advantages over conventional exercise.

## Conclusions

Yoga is a promising, lower-burden alternative rather than a definitively superior intervention, particularly in contexts where time constraints limit access to supervised exercise. High adherence (>90%) and the absence of SAEs support the safety and acceptability of both programs when delivered with appropriate supervision and individualized modification. Larger, multicentered, dose-matched trials with longer follow-up are needed to determine the durability of effects and confirm whether yoga offers sustained, meaningful advantages over conventional exercise-based care. Until such data are available, yoga may be considered an effective component of multimodal, guideline-based care for CMLBP, especially for patients seeking time-efficient, nonpharmacological options.
